# Supplementary material for: Seroprevalence of Leptospira spp. Infection in Cattle from Central and Northern Madagascar
Source: Int J Environ Res Public Health. 2019 Jun 6;16(11):2014. doi: 10.3390/ijerph16112014 (PMC6603958; doi:10.3390/ijerph16112014)
Supplement: Supplementary file 1 [file ijerph-16-02014-s001.zip › Table S2.docx]

**Table S2.** Number of individuals and relative prevalence per *Leptospira* spp. serovar. Serovars included in the test: *L.*Tarassovi (T), *L.*Hardjo (HJ), *L.*Grippotyphosa (G), *L.*Pomona (P), *L.*Autumnalis (AT), *L.*Pyrogenes (PY), *L.*Bataviae (BAT), *L.*Australis (A), *L.*Javanica (JAV), *L.*Ballum (BA), *L.*Canicola (CAN) and *L.*Icterohaemorrhagiae (ICT).

| **MAT titer** |  | **All serovars** | | **T** | **HJ** | **G** | **P** | **AT** | **PY** | **BAT** | **A** | **JAV** | **BA** | **CAN** | **ICT** |
| --- | --- | --- | --- | --- | --- | --- | --- | --- | --- | --- | --- | --- | --- | --- | --- |
| 1:100 | n  % | | 81  41.8 | 31  16.0 | 11  5.7 | 9  4.6 | 11  5.7 | 6  3.1 | 5  2.6 | 5  2.6 | 2  1.0 | -  - | 1  0.5 | -  - | -  - |
| 1:200 | n  % | | 62  32.0 | 33  17.0 | 9  4.6 | 7  3.6 | 3  1.6 | 4  2.1 | 3  1.6 | 1  0.5 | -  - | 2  1.0 | -  - | -  - | -  - |
| 1:400 | n  % | | 20  10.3 | 13  6.7 | 5  2.6 | 2  1.0 | -  - | -  - | -  - | -  - | -  - | -  - | -  - | -  - | -  - |
| 1:800 | n  % | | 5  2.6 | 1  0.5 | 2  1.0 | -  - | 1  0.5 | -  - | 1  0.5 | -  - | -  - | -  - | -  - | -  - | -  - |
| 1:1600 | n  % | | 1  0.5 | -  - | -  - | 1  0.5 | -  - | -  - | -  - | -  - | -  - | -  - | -  - | -  - | -  - |
| 1:3200 | n  % | | -  - | -  - | -  - | -  - | -  - | -  - | -  - | -  - | -  - | -  - | -  - | -  - | -  - |
| 1:6400 | n  % | | -  - | -  - | -  - | -  - | -  - | -  - | -  - | -  - | -  - | -  - | -  - | -  - | -  - |
