# Supplementary material for: Seroprevalence of Leptospira spp. Infection in Cattle from Central and Northern Madagascar
Source: Int J Environ Res Public Health. 2019 Jun 6;16(11):2014. doi: 10.3390/ijerph16112014 (PMC6603958; doi:10.3390/ijerph16112014)
Supplement: Supplementary file 1 [file ijerph-16-02014-s001.zip › Table S1.docx]

**Table S1.** Seroprevalence of each serovar according to geographic sampling region.

| **Serovar** | **Bongolava**  **%** | **Haute Matsiatra**  **%** | **Menabe**  **%** | **Sofia**  **%** | **Vakinankaratra**  **%** | ***p*-value** |
| --- | --- | --- | --- | --- | --- | --- |
| *L.* Tarassovi  *L.* Hardjo  *L.* Grippotyphosa  *L.* Pomona  *L.* Autumnalis  *L.* Pyrogenes  *L.* Bataviae  *L.* Australis  *L.* Javanica  *L.* Ballum  *L.* Canicola  *L.* Icterohaemorrhagiae | 32.9  13.2  13.2  10.5  9.2  5.3  1.3  1.3  2.6  0.0  0.0  0.0 | 48.4  14.5  12.9  3.2  1.6  4.8  4.8  1.6  0.0  0.0  0.0  0.0 | 36.0  12.0  0.0  0.0  4.0  8.0  4.0  0.0  0.0  0.0  0.0  0.0 | 38.1  19.1  4.8  19.1  4.8  0.0  4.8  0.0  0.0  0.0  0.0  0.0 | 50.0  0.0  0.0  10.0  0.0  0.0  0.0  0.0  0.0  10.0  0.0  0.0 | 0.29  0.93  0.45  0.10  0.17  0.75  0.73  0.97  -  -  -  - |
